# From follicles to fear: how hair transplantation trends in Brazil highlight the risks of hormone overuse

**DOI:** 10.1590/1806-9282.20242072

**Published:** 2025-10-17

**Authors:** Hudson Dutra Rezende

**Affiliations:** 1 Centro Universitário Lusíada – Santos (SP), Brazil.

Hormone replacement therapy (HRT) offers numerous well-documented benefits for patients with legitimate medical indications^1,2^. It has long been utilized by medical professionals to enhance patients’ quality of life, including improvements in libido, sexual function, energy levels, muscle mass, and bone density^2^. However, a critical question remains: how many patients undergoing hormonal therapy are truly in medical need? This issue demands careful clinical evaluation, particularly given that a significant portion of the population uses testosterone and related stimulants without proper medical supervision. Furthermore, some patients are subjected to excessive and irrational hormone use by physicians who prioritize aesthetics over health, often overlooking the risks of long-term complications. In some cases, underlying psychological conditions such as body dysmorphic disorder may drive patients to seek hormonal treatments or hair restoration procedures, highlighting the need for thorough mental health evaluation alongside medical care.

The conflict between hair restoration and HRT is clear from a physiological standpoint. While testosterone and its metabolites enhance physical appearance and performance, they exert a detrimental effect on the hair cycle, leading to long-term miniaturization and the development of androgenetic alopecia (AGA) in predisposed individuals^3^. This explains why dermatologists and physicians specializing in hair transplantation frequently encounter young patients seeking hair restoration while simultaneously undergoing HRT. This scenario represents a dilemma for both patients and physicians, as dihydrotestosterone, a metabolite of testosterone, enhances physical performance while inducing hair follicle miniaturization, exacerbating AGA^3^.

Clinical practice demonstrates a growing demand for hair restoration procedures. According to The Practice Census 2022 conducted by the International Society of Hair Restoration Surgery, over 703,000 hair restoration surgeries were performed globally in 2021, marking a 250% increase compared to 2010, when approximately 279,000 procedures were recorded^4^. In Brazil, this trend is corroborated by data from the Brazilian Association of Hair Restoration Surgery, which highlights that hair transplantation has consistently dominated Google searches related to transplant procedures since 2004, accounting for 48.43% of these queries^5^.

Similarly, the increasing use of HRT parallels the rising demand for hair restoration, prompting medical organizations to issue warnings about the indiscriminate and inappropriate use of hormonal therapies. For instance, on April 11, 2023, the Federal Council of Medicine in Brazil issued a resolution restricting the use of androgenic steroids and anabolic agents for aesthetic purposes, muscle gain, and athletic performance enhancement. This measure was supported by the Brazilian Society of Dermatology and other medical entities, which had already been raising awareness about the risks associated with the improper use of these substances. Additionally, on October 18, 2024, the Brazilian Health Regulatory Agency (Anvisa) suspended the production, commercialization, promotion, and use of hormonal implants, commonly referred to as "beauty chips." This decision followed reports from medical organizations regarding the growing misuse of these implants in the country.

For patients using steroids for aesthetic or athletic purposes without medical supervision, the long-term consequences remain uncertain but are expected to raise significant concerns. What are the risks associated with long-term testosterone use? What dangers exist for individuals who continuously renew their "beauty chips"? The answers to these questions remain unclear. While HRT can offer considerable benefits for some individuals, it may lead to various complications for others^1,6–9^ (Table 1). Furthermore, some patients deny improper hormone use or conceal their replacement regimens, thus avoiding strict medical guidance and complicating their care. According to Baron et al., patients under exogenous androgen replacement typically present with elevated hematocrit levels, undetectably low serum luteinizing hormone levels, and reduced sex hormone-binding globulin, which can aid in clinical evaluation and the development of more effective medical strategies.

**Table 1:** Common hormones used for muscle gain and their tegumentary adverse effects.

Hormone/substance	Mechanism of action	Tegumentary adverse effects
Testosterone (and derivatives)	Androgen receptor agonist; increases protein synthesis and muscle mass	Acne, seborrhea, androgenetic alopecia, hirsutism (in women), and increased skin oiliness
Stanozolol	Synthetic anabolic steroid; androgenic and anabolic action	Severe acne, hair loss, skin texture changes, and striae
Oxandrolone	Mild anabolic steroid; low aromatization; increases strength and lean mass	Acne, alopecia, oily skin, gynecomastia (less common), and hirsutism (in women)
Trenbolone	Potent androgen; strong binding to androgen receptor	Cystic acne, accelerated hair loss, excessive sweating, strong body odor, and oily skin
Dianabol (methandrostenolone)	Androgen with high aromatization; promotes water retention and rapid strength gains	Inflammatory acne, gynecomastia, oily skin, and striae
Clomiphene/tamoxifen	Selective estrogen receptor modulators (SERMs); stimulate endogenous testosterone	Mild: dry skin and subtle skin changes
Anastrozole	Aromatase inhibitor; reduces the conversion of androgens to estrogens	Dry skin, hair thinning, and increased skin sensitivity
Growth hormone (GH)	Stimulates cell growth; promotes lipolysis and anabolism	Hyperhidrosis, skin thickening, acne, oily skin, and carpal tunnel syndrome
Insulin (illicit use)	Stimulates glucose uptake and protein synthesis	Local lipodystrophy, excessive sweating, and oily skin
Thyroid hormones (T3)	Increase basal metabolism; promote fat loss	Excessive sweating, thin/warm skin, hair loss, and brittle nails

From a dermatological perspective, patients seeking treatment for active or uncontrolled AGA—despite adhering to appropriate dermatological therapies—should also have their hormonal profiles assessed. While some individuals with so-called refractory AGA may not be undergoing direct HRT, they might unknowingly consume substances with androgen-stimulating properties. These substances are often prescribed in compounded formulations containing multiple ingredients, such as tamoxifen, clomiphene, and anastrozole. Patients frequently report being inadequately informed about these compounds’ intended purposes or, more critically, their associated risks. In such cases, baldness often becomes a minor medical concern, as other complications—including cardiovascular issues, gynecomastia, acne, tendon ruptures, elevated liver enzymes, erythrocytosis, and neuropsychiatric symptoms (such as mood disorders and aggression)—take precedence^1,6–8^.

Finally, the increasing demand for hair transplantation offers Brazilian physicians an opportunity to observe emerging trends in hormonal treatment, as the population diagnosed with AGA often overlaps with those undergoing HRT. Ongoing medical vigilance is essential to guide patients through their parallel treatments, whether cardiological, dermatological, neurological, or otherwise. This ensures that patients can make informed decisions regarding the risks they choose to take.

## Data Availability

The datasets generated and/or analyzed during the current study are available from the corresponding author upon reasonable request.

## References

[1] Margolese RG, Cecchini RS, Julian TB, Ganz PA, Costantino JP, Vallow LA (2016). Anastrozole versus tamoxifen in postmenopausal women with ductal carcinoma in situ undergoing lumpectomy plus radiotherapy (NSABP B-35): a randomised, double-blind, phase 3 clinical trial. Lancet.

[2] Yabluchanskiy A, Tsitouras PD (2019). Is testosterone replacement therapy in older men effective and safe?. Drugs Aging.

[3] Nestor MS, Ablon G, Gade A, Han H, Fischer DL (2021). Treatment options for androgenetic alopecia: efficacy, side effects, compliance, financial considerations, and ethics. J Cosmet Dermatol.

[4] International Society of Hair Restoration Surgery (2022). 2022 practice census results.

[5] Brazilian Association of Hair Restoration Surgery (2024). Hair restoration trends and statistics in Brazil.

[6] Ye L, Zhong F, Sun S, Ou X, Yuan J, Zhu J (2023). Tamoxifen induces ferroptosis in MCF-7 organoid. J Cancer Res Ther.

[7] Kapoor J, Singh N, Agrawal N, Ahmed R, Bhurani D (2019). A case of anastrozole-induced erythrocytosis. Indian J Hematol Blood Transfus.

[8] Wei M, Xu YR, Liu K, Wen P (2020). Anastrozole-induced pulmonary cryptococcosis in a patient with early breast cancer: a case report. Medicine (Baltimore).

[9] Kim YJ, Cohen PR (2020). Anastrozole-induced dermatitis: report of a woman with an anastrozole-associated dermatosis and a review of aromatase inhibitor-related cutaneous adverse events. Dermatol Ther (Heidelb).

